# Arizona bark scorpion venom resistance in the pallid bat, *Antrozous pallidus*

**DOI:** 10.1371/journal.pone.0183215

**Published:** 2017-08-30

**Authors:** Bradley H. Hopp, Ryan S. Arvidson, Michael E. Adams, Khaleel A. Razak

**Affiliations:** 1 Graduate Neuroscience Program, University of California, Riverside, California, United States of America; 2 Departments of Entomology and Cell Biology & Neuroscience, University of California, Riverside, California, United States of America; 3 Department of Psychology, University of California, Riverside, California, United States of America; Instituto Butantan, BRAZIL

## Abstract

The pallid bat (*Antrozous pallidus*), a gleaning bat found in the western United States and Mexico, hunts a wide variety of ground-dwelling prey, including scorpions. Anecdotal evidence suggests that the pallid bat is resistant to scorpion venom, but no systematic study has been performed. Here we show with behavioral measures and direct injection of venom that the pallid bat is resistant to venom of the Arizona bark scorpion, *Centruroides sculpturatus*. Our results show that the pallid bat is stung multiple times during a hunt without any noticeable effect on behavior. In addition, direct injection of venom at mouse LD_50_ concentrations (1.5 mg/kg) has no effect on bat behavior. At the highest concentration tested (10 mg/kg), three out of four bats showed no effects. One of the four bats showed a transient effect suggesting that additional studies are required to identify potential regional variation in venom tolerance. Scorpion venom is a cocktail of toxins, some of which activate voltage-gated sodium ion channels, causing intense pain. Dorsal root ganglia (DRG) contain nociceptive neurons and are principal targets of scorpion venom toxins. To understand if mutations in specific ion channels contribute to venom resistance, a pallid bat DRG transcriptome was generated. As sodium channels are a major target of scorpion venom, we identified amino acid substitutions present in the pallid bat that may lead to venom resistance. Some of these substitutions are similar to corresponding amino acids in sodium channel isoforms responsible for reduced venom binding activity. The substitution found previously in the grasshopper mouse providing venom resistance to the bark scorpion is not present in the pallid bat, indicating a potentially novel mechanism for venom resistance in the bat that remains to be identified. Taken together, these results indicate that the pallid bat is resistant to venom of the bark scorpion and altered sodium ion channel function may partly underlie such resistance.

## Introduction

Animal venoms used for predation, defense and/or intraspecific competition are typically a complex mixture of toxins that can cause intense pain, tissue damage and death. Given the relative abundance of venomous species across phyla, it is not surprising that various predators and prey of venomous animals have developed resistance to one or more of these toxins [1–8]. There are two fundamentally important reasons for studying venom resistance. First, mechanisms of pain modulation can be identified with potential utility in human pain management. These studies will provide insights on how excitability of neurons can be adaptively modified by changes in ion channel sequences. Second, a comparison across species will provide insights into different mechanisms of venom resistance, including evolution of ion channel and receptor modifications and blood serum based mechanisms [1–8]. In this study, we present evidence that the pallid bat (*Antrozous pallidus*) is resistant to venom of the Arizona bark scorpion (*Centruroides sculpturatus*), North America’s most venomous scorpion. Transcriptome analysis of bat dorsal root ganglia (DRG) was employed to identify potential mechanisms that may contribute to such resistance.

Bats use a variety of foraging strategies. The most common strategy amongst insectivorous bats is ‘aerial hawking’ wherein echolocation is used to detect, localize and hunt prey in flight. Another strategy, observed in a small group of bat species across families, is known as ‘gleaning’. Gleaning bats use a combination of echolocation and passive hearing of prey-generated noise to hunt prey from various substrates. The pallid bat is a gleaner, depending extensively on prey-generated noise (rustling, walking, etc.) to hunt terrestrial prey, while echolocation is used mostly for obstacle avoidance and general orientation [9]. Pallid bats localize prey-generated noise and land on or near potential prey. This foraging strategy puts the pallid bat in close proximity to scorpions.

Numerous scorpion genera are sympatric with the pallid bat, the most venomous being *Centruroides* [10]. This includes the Arizona bark scorpion (*C*. *sculpturatus*), whose sting induces extreme pain and occasionally death in humans [11]. Observations of night roosts indicate that pallid bats consume various species of scorpions including members of the *Centruroides* genus [9, 12–15]. Anecdotal evidence suggests that the pallid bats hunt and consume Arizona bark scorpions, but whether they simply avoid stings or are resistant to effects of the venom is unclear. If the latter, the pallid bat would provide an opportunity to determine mechanisms of venom resistance and pain modulation. In addition, studies of the pallid bat would provide comparative insights on mechanisms of venom resistance, given that at least one mechanism of Arizona bark scorpion venom resistance is known in the grasshopper mouse (*Onychomys torridus*) [16].

The first aim of this study was to use high-speed video to determine if Arizona bark scorpions sting the pallid bat during predation. Given the potential variability in the amount of venom delivered by a bark scorpion in a hunt, the second aim was to inject a known concentration of Arizona bark scorpion venom directly into the pallid bat. For comparative purposes, the same concentration was injected in mice. Upon determination that the pallid bat is indeed resistant to bark scorpion venom, we initiated the third aim: exploring possible molecular mechanisms of resistance. To this end, we performed a transcriptome analysis of pallid bat dorsal root ganglia (DRG). Although multiple mechanisms of venom resistance have been identified across species [1, 3, 6, 7, 17, 18], we focused here on sequencing voltage sodium channels for two main reasons. First, these ion channels are principal targets of bark scorpion venom and mutations in these channels are known to confer resistance to venom. Second, we wanted to determine if the grasshopper mouse and the pallid bat have converged on similar mechanisms for venom resistance. Many sequence motifs in voltage gated sodium channels are important for venom toxin binding (alpha toxin binding sites:[19–23] beta toxin binding sites: [24–28], review [29]). The rationale for the third aim was to identify substitutions in pallid bat DRG that potentially confer resistance to the painful effects of Arizona bark scorpion venom. Previous studies of grasshopper mouse sodium channels revealed that a switch of a glutamine and a glutamate in IIIS5-S6 of Nav1.8 was sufficient for resistance to bark scorpion venom [16]. An important goal of this study was to determine if the same mechanism of resistance is observed in the pallid bat Nav1.8. We found that the pallid bat is resistant to Arizona bark scorpion venom and describe amino acid substitutions in voltage gated sodium ion channels (Nav 1.7 and 1.8) in the DRG that may confer such resistance. However, the mutation described in the grasshopper mouse is not found in the pallid bat, suggesting a potentially novel mechanism of pain modulation.

## Materials and methods

### Animal husbandry

This study was carried out in strict accordance with the animal welfare guidelines of the National Institutes of Health and the Institutional Animal Care and Use Committee (IACUC) at the University of California, Riverside. The protocol was reviewed and approved by the IACUC. Pallid bats, mist-netted in Arizona, New Mexico and California, were housed on a reversed 12:12 light:dark cycle in an 11 x 14 ft^2^ room, which allowed them to fly freely. Bats were obtained using scientific collecting permits issued by each of these states. Crickets and/or mealworms and water were supplied *ad libitum*. Food was withheld 24 hours before encounters with scorpions to ensure motivation to hunt. Scorpions were purchased from Scorpion Sweepers, LLC (Scottsdale, AZ).

### Bat-scorpion encounters

Scorpion-bat interactions were filmed in a behavior room (13 x 14 x 8 cu. ft.) in which the scorpion was placed in an open top box (3 x 3x 4 cu.in.) and the bat performed a detect, land and hunt task. No training was required because this is a natural behavior. Additional filming environments included an empty terrarium (1.5 x 0.5 x 1 cu. ft.) with ~1” soil covering the bottom. After the scorpion was in the aquarium for a few minutes, a pallid bat was placed in the same enclosure. The aquarium was chosen to constrain high speed filming to a limited area to record the interaction in more detail. A Canon XA10 video camera and Phantom high-speed camera were positioned to capture the interaction to determine if the pallid bat was stung during the attack. For quantification purposes, a ‘sting’ is defined as any time the aculeus tip touched the bat.

### Venom injection

Freeze dried *C*. *sculpturatus* venom was obtained from Spider Pharm (Yarnell, AZ) and kept at -80°C until use. Venom was diluted in saline 1–2 hours before injection. To ensure venom toxicity and to obtain more detailed behavioral response quantification than is currently available in the literature [30, 31], venom was injected into mice (n = 4 at 1.0 mg/kg b.w.) in the range of LD_50_ previously established for *C*. *sculpturatus* [30, 31]. While previous investigators [16] injected venom into the soft tissue of the paw, pallid bat limb extremities have very little soft tissue. To maintain consistent injection sites across mice and bats, the area between the scapulae was chosen for venom injection. As a control, on the day before venom injection, each mouse was injected subcutaneously between the scapulae with 30 μl saline and observed. After saline or venom injection, mice were observed for up to 10 minutes, and signs of pain were quantified. Behaviors associated with pain were quantified as the number of whole body jerky movements (convuslions), vocalizations and time spent grooming. Venom was injected into bats (n = 13) according to the same protocol. A venom dose of 1.0 mg/kg was used in 2/13 bats, 1.5 mg/kg was used in 7/13 bats and 10 mg/kg was used in 4/13 bats. Bats were observed for one hour following venom injection and then placed in a cage in the colony room and observed periodically for an additional 24–48 hours before being released back in the colony room. Humane endpoints were established for both mice and bats. Mice were to be euthanized with sodium pentobarbital (100–125 mg/kg b.w., i.p.) at the end of the 10 minute observation period or if the total time the mouse exhibited abnormal behaviors (convulsions, immobility or prostration) was longer than 1 minute. For bats as well, a 10 minute observation period was used to study effects of venom. Bats that showed abnormal behaviors for more than 10 minutes after injection were to be euthanized with sodium pentobarbital (100–125 mg/kg b.w., i.p.). If euthanasia was not necessary because the effects were minimal or non-existant, buprenorphine (0.05–0.1 mg/kg) was injected after the 10 minute observation window. As described in the Results section below, none of the bats were euthanized because the effects, if present, were transient. There were no deaths prior to the 10 minute observation periods for mice because the injected dose was less than the known LD_50_. There were no deaths in bats because as we report below, venom only had transient effects and that too only in 2/13 bats. A fatal injection of sodium pentobarbital was used to euthanize all four mice within the 10 minute observation period.

#### RNA extraction and transcriptome methods

Dorsal root ganglia (DRG) were extirpated from two pallid bats following a fatal dose of sodium pentobarbital (125 mg/kg b.w.). Cervical and thoracic DRGs were placed immediately in TRIzol and homogenized. Total RNA was purified with PureLink RNA mini kit (Ambion) according to manufacturer’s instructions. Agilent Bioanalyzer was used to assure the quality of the RNA and only samples with a RNA Integrity Number (RIN) greater than 9 was accepted for sequencing. RNAseq libraries were made using NEBNext Ultra Directional RNA Library Prep kit for Illumina (New England Biolabs; Ipswich, MA) (prepared by the Institute of Integrative Genome Biology at University of California, Riverside) and libraries were multiplexed and run on the same lane of a NextSeq RNA sequencer (Illumina).

Resultant reads were assembled using the TRINITY [32] software pipeline with custom settings (S1 Fig). The software Benchmarking Universal Single-Copy Orthologues (BUSCO) [33] was run to assess the assembly was complete (76%-80% of all BUSCOs found) suggesting the assembly captured most genes expressed in DRG. Putative open reading frames (ORF) were extracted from the transcriptome assembly via the TransDecoder plugin for Trinity. ORFs were then aligned to two databases using BLAST, one database constructed from genes from *Myotis lucifugus*, *Myotis davidii*, *Myotis brandtii*, *and Pteropus alecto* (referred to as 4 Bats Database) and the other from the Swiss-Prot database. Duplicate gene hits were eliminated by keeping the hit with the lowest e-value as the pallid bat gene. If two ORFs had the same e-value, then the ORF with the higher fragments per kilobase of exon per million fragments mapped (FPKM) value was chosen as the representative isoform, referred to as Unique Gene Hit. An overview of the assembly and quality control can be found in S1 Table. Sequences of Nav1.7 and Nav1.8 were then compared to other species using Clustal Omega [34] and Jalview [35].

## Results

### Bat-scorpion predator-prey encounters

Pallid bats were video-recorded attacking Arizona bark scorpions to determine whether bats are stung or avoid stings. Next we injected Arizona bark scorpion venom directly into pallid bats to determine resistance of pallid bats to the venom.

AZ bark scorpions stung pallid bats, sometimes multiple times, during a hunt. Five high-speed video recordings provided a clear view of the scorpion behavior during pallid bat attacks (see S1 Video for an example). Table 1 provides analysis of pallid bat attacks and scorpion defense, scored as number of stings. Bats 1, 3–5 consumed the scorpion at the end of the encounter, demonstrating that pallid bats eat Arizona bark scorpions. None of the bats reacted to stings during or after the encounter. Bat 2 abandoned the attack, likely because the aculeus became caught in the bat’s lip and caused injury likely unrelated to venom injection. Observation of this bat after the encounter showed no behavioral response to envenomation. These videos clearly show that the aculeus contacts the pallid bat multiple times during a hunt. It is presumed that venom was injected in at least some of these instances. However, we observed no mortality, morbidity, or noticeable effect on behavior. It did not appear that the bat was specifically trying to grab the scorpion in any specific manner that prevented aculeus contact.

**Table 1:** Time required for bats to subdue scorpions or abandon attack and the number of observed stings during each encounter.

	Bat 1	Bat 2	Bat 3	Bat 4	Bat 5
**Length of Encounter in Sceonds**	6.02	1.42	2.5	1	4.13
**# Stings**	3	1	10*	1	4

*During this trial, the scorpion aculeus was oriented on or near the bat head for most of encounter, resulting in many aculeus-bat contacts that may not have been genuine stings.

### Venom injection

Tables 2 and 3 describe venom injection experiments in mice and pallid bats, respectively. All four mice showed behavioral signs of envenomation (Table 2). These included intense grooming, particularly of the face, and vocalizations, convulsions and disoriented movements. These behaviors were not seen following saline injections. Likely because the concentration tested was less than reported LD_50_, none of the mice died during the first 10 minutes of post-injection observation. However, altered behaviors were consistent and obvious even at the 1 mg/kg venom dose.

**Table 2:** Behavioral responses of mice following scorpion venom injection.

	Dose (mg/kg)	Number of convulsions	Time spent grooming (min:sec)	Number of audible vocalizations	Time to first convulsion (min:sec)	Time to first grooming (min:sec)
M1	1	19 (0)	6:17 (1:13)	0 (0)	1:19 (N/A)	1:38 (0:50)
M2	1	60 (0)	7:43 (0:49)	21 (0)	2:49 (N/A)	2:37 (1:35)
M3	1	46 (0)	6:30 (0:56)	35 (0)	4:14 (N/A)	5:09 (1:40)
M4	1	43 (0)	5:45 (0:34)	36 (0)	5:29 (N/A)	4:15 (2:47)

Responses to saline injection are shown in parenthesis.

**Table 3:** Dose and indication of response to Arizona bark scorpion venom when injected into pallid bats.

Animal	Venom Dose mg/kg	Number of convulsions	Time spent vocalizing (seconds)	Time Spent walking backward
Bat_1	1	0	0	0
Bat_2	1	0	0	0
Bat_3	1.5	0	0	0
Bat_4	1.5	0	0	0
Bat_5	1.5	0	0	0
Bat_6	1.5	0	0	0
Bat_7	1.5	0	0	0
Bat_8	1.5	0	0	0
Bat_9	1.5	0	39	114
Bat_10	10	22	0	0
Bat_11	10	1	0	0
Bat_12	10	0	0	0
Bat_13	10	0	0	0

For 11/13 bats, no observable behavioral modifications were present following injection. Convulsions are defined as whole body jerky movements. Unlike the mouse, venom induced grooming was absent in bats.

For eight out of nine bats injected with 1 or 1.5 mg/kg dose, venom did not produce noticeable effects on behavior (Table 3). One out of nine injected bats (Bat 3) produced audible vocalizations and lumps on its snout that appeared to be an allergic reaction. Backward walking was also elicited following injection. Vocalizations and backward walking were absent after 10 minutes. At the highest dose tested (10 mg/kg), 3/4 bats showed no noticeable effects. However, one of the bats showed abnormal jerky movements for the first 7 minutes. None of the bats showed any effects after 10 minutes. Taken together, these data indicate that almost all pallid bats tested were resistant to Arizona bark scorpion venom at doses up to 10 mg/kg, with the possibility of reactions in some bats that cannot be fully discounted.

### Transcriptome analysis

Assembly of raw Illumina reads were separated into two groups based on biological replicates and labeled DRG1 and DRG2. Assembly is summarized in Supplementary Materials (S1 Table). The N50 for both samples was ~1500 and individual voltage-gated sodium ion channels were examined to ensure full-length transcripts were present. The software BUSCO was run to assess if the assembly was complete (76%-80% of all BUSCOs found). TransDecoder extracted 94,522 ORFs from DRG1 assembly and 109,948 from DRG2 assembly which yielded approximately 24,500 unique gene hits per tissue sample when processed in our BLAST pipeline.

### Sequence analysis of voltage gated sodium ion channels

**Fig 1:**
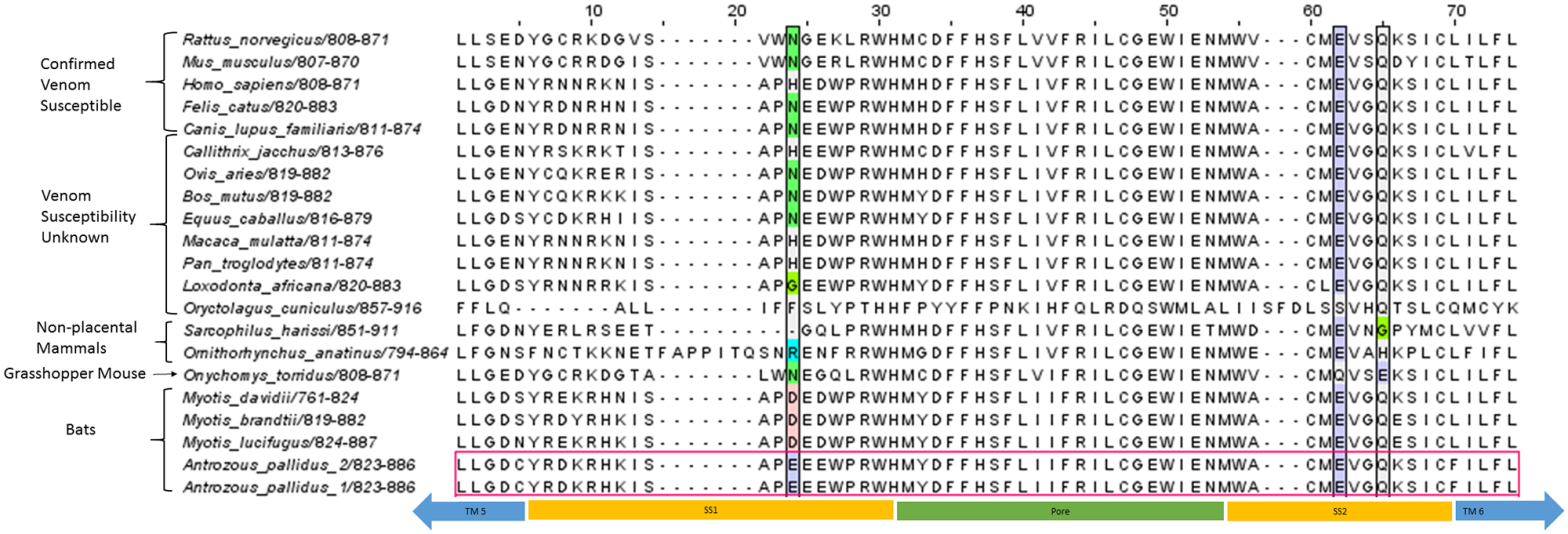
Alignment of extracellular region IIS5-S6 of Nav1.8. Colored columns in SS2 show residues important for granting venom resistance in grasshopper mouse. Most species have a glutamate at position 62 in the alignment shown and a glutamine at position 65, including the pallid bat. However, the grasshopper mouse has these two amino acids switched. This switch has been shown to confer venom resistance in this species [16].

Transcriptome analysis revealed three voltage-gated sodium channels expressed in pallid bat DRG: Nav1.7, Nav1.8, and Nav1.9. Due to limited information on the effect of scorpion venom on Nav1.9, it was not further analyzed. A recent study of the grasshopper mouse reported substitutions of glutamate and glutamine in domain 2 of Nav1.8, which enhance binding affinity of Arizona bark scorpion toxins leading to channel block [16]. Since Nav1.8 is necessary for action potential propagation, block of Nav1.8 functions effectively as an analgesic, shutting down the pain-signaling pathway [16]. To determine if venom resistance seen in the pallid bat can be attributed to the same mutations, we analyzed domain 2 of Nav1.8. Sequence data indicates that this mechanism does not operate in the pallid bat (Fig 1, S2 Fig shows additional comparative details of pallid bat Nav1.8 sequence in domains known to be important for venom binding in Nav1.7). Indeed, the pallid bat sequence in this region is identical to that of humans and other species susceptible to scorpion venom. Thus, the pallid bat likely has a novel mechanism for Arizona bark scorpion venom resistance.

**Fig 2:**
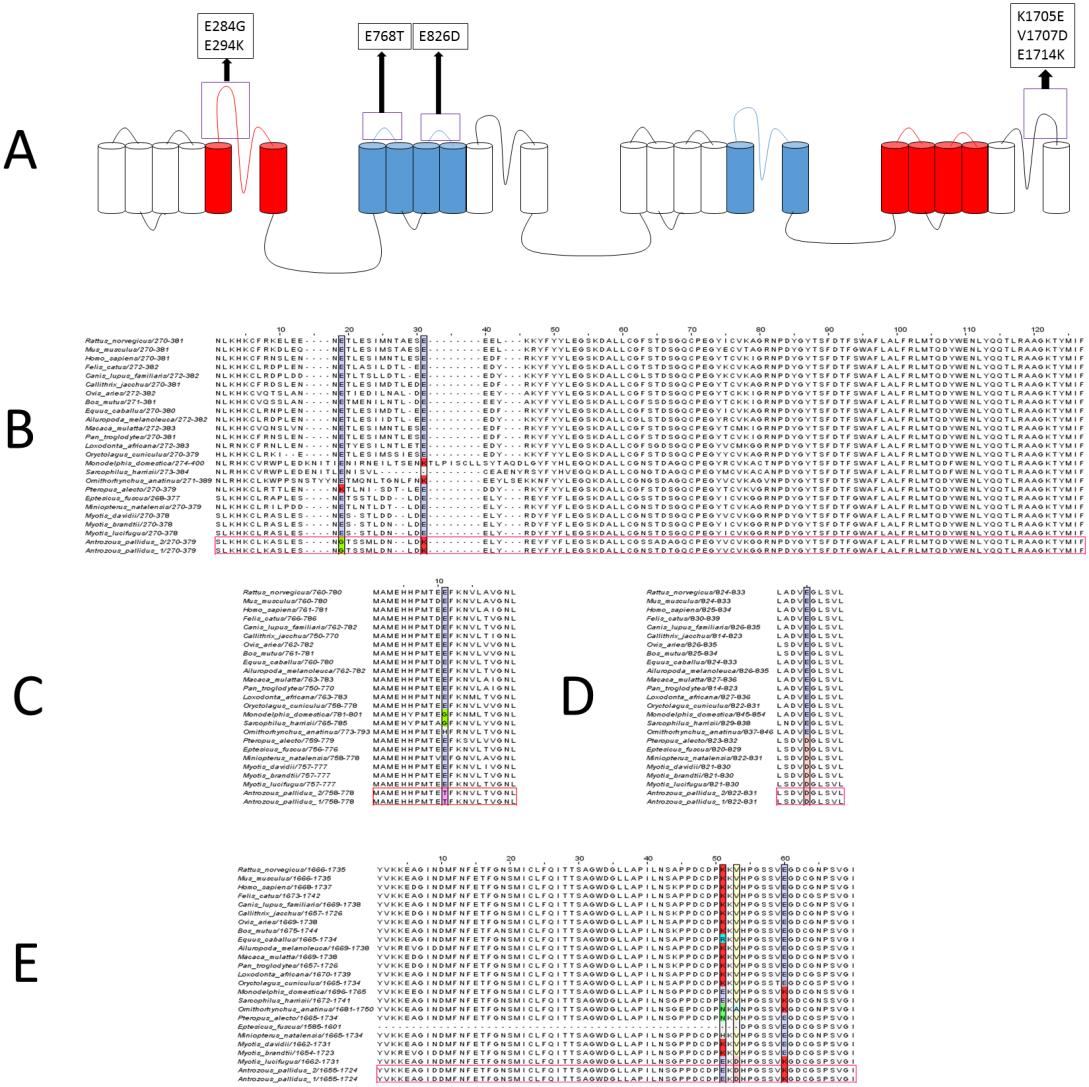
Comparison of selected extracellular loops in Nav1.7 known to be involved in scorpion toxin binding. While Nav1.7 displays normal activity in the grasshopper mouse, it may be altered in the pallid bat providing venom resistance. (A) Schematic of Nav1.7 showing known scorpion toxin binding regions and regions of special note in the pallid bat; red are known alpha scorpion toxin binding regions and blue are known beta scorpion toxin binding regions. (B) Extracellular IS5-S6. (C) Extracellular region IIS1-S2. (D) IIS3-S4. (E) Extracellular region IVS5-S6.

Nav1.7 is the main target of scorpion venom toxins [36–38]. Fig 2A shows known scorpion toxin binding regions in extracellular regions of sodium channels [19–25, 27, 28, 39, 40]. Fig 2B–2E shows alignments of selected extracellular regions of Nav1.7 across various species; colored regions are locations where the pallid bat either has an amino acid substitution known to be important for venom binding in other sodium channel isoforms or has a significant change in amino acid chemistry. Special attention is given to changes in acidic residues, as they are crucial for toxin binding [21, 23, 41]. Substitutions of special note are described in Fig 2.

### Availabilty of sequences

The sequences for SCN10a (Nav1.8) and SCN9a (Nav1.7) have been deposited in GenBank

Nav1.7 (GenBank Accession MF616470) and Nav1.8 (GenBank Accession MF616471)

## Discussion

Observations of bat-scorpion interactions indicate that the pallid bat is stung while hunting the Arizona bark scorpions. In all but one instance, the bat successfully killed and ate the scorpion with the exception most likely attributable to mechanical damage caused by the scorpion. Moreover, direct injection of venom at a dose known to induce strong pain responses in mice caused no evident pain responses in eight out of the nine bats tested. Of the additional four bats tested with 10 mg/kg venom, one bat displayed a transient behavioral reaction that lasted less than 10 minutes. The other bats were unaffected. We interpret these data to mean that the pallid bat is resistant to Arizona bark scorpion venom. Because at least two bats showed a reaction, albeit transiently, the possibility of regional variation in venom tolerance [25] cannot be discounted based on the current study. Grasshopper mice populations that are sympatric with the Arizona bark scorpion exhibit a higher LD_50_ (~18 mg/kg) compared to populations that are parapatric (~12 mg/kg) or allopatric (~10 mg/kg) [42]. Given variation in sympatry between the pallid bat and *C*. *sculpturatus*, future studies with different popluations of pallid bats [43] and additional venom doses are required to evaluate population differences in venom tolerance.

One mechanism underlying venom resistance in the grasshopper mouse is known [16]. An amino acid substitution in the Nav1.8 sodium ion channel (Fig 1) causes the venom to act as an analgesic by inactivating pain sensing neurons of the DRG. Sequence analysis showed that this mechanism is not present in the pallid bat, suggesting that this form of venom resistance occurs through a different, hitherto unknown mechanism. Pallid bat sodium channel sequences show several substitutions in toxin binding regions that may contribute to resistance. A number of these changes involve acidic residues. While studies [21,23,40,41] have shown that changing acidic residues in various isoforms of sodium channels alters toxin binding, little work has focused on voltage-gated sodium channels in DRG (Nav1.7, Nav1.8, and Nav1.9). However, given that acidic residues are important in toxin binding to isoforms of Nav1.7, it is possible that substitutions involving acidic amino acid side chains observed in the pallid bat alter binding affinity of scorpion venom toxins.

In Nav1.7, we see various substitutions that are either pallid bat specific, bat specific, or bat and non-placental mammal specific. For example, Fig 2B shows the known venom binding region in the extracellular region of domain 1 between TM 5 and 6 (IS5-S6) [27], where the pallid bat has an E284G (pallid bat numbering) substitution with respect to venom-susceptible species. The only other species examined that does not have a glutamate in this location is the black flying fox (*Pteropus alecto)*, which has a lysine. Also in IS5-S6 the pallid bat has an E294K substitution. All other species examined have a glutamate at this position with the exception of the three non-placental mammals: Gray short-tailed opossum (*Monodelphis domestica)*, Tasmanian devil *(Sarcophilus harrisii)*, and Duckbilled platypus (*Ornithorhynchus anatinus)*. Both the opossum and platypus have a glutamate to lysine substitution, while the Tasmanian devil has a deletion in this region. The change in charge between the pallid bat and two non-placental mammals may indicate convergent evolution of venom resistance in these three species. However, it is unclear if the opossum and platypus are scorpion venom resistant. Scorpion venom resistance of non-placental mammals is in general unclear, but all have overlapping ranges with venomous species. Snake venom resistance is reported in other species of opossum [1] and arthropods are a known prey item of gray short-tailed opossum. The Tasmanian devil is a known generalist predator whose diet includes arthropods and venomous snakes [44]; however its venom resistance status is unknown. The platypus employs venom for intraspecific mate competition [45]. Given the high potency of this venom in humans, platypuses most likely possess some level of resistance to their own venom. In non-placental mammals, sodium channel sequence similarities to the pallid bat in scorpion toxin binding regions suggests they have a mechanism of venom resistance similar to that of the pallid bat.

In IIS1-S2, we see that the pallid bat again shares more sequence similarity with non-placental mammals. The pallid bat E769T substitution contrasts with marsupial glycine and platypus histidine substitutions. The investigators in [40], showed that changing glutamate to either a glutamine or cysteine greatly reduces the binding affinity of the beta scorpion toxin CssIV to Nav1.2 and we may be seeing a similar toxin binding altering substitution in the pallid bat Nav1.7

One intriguing result of the comparative analysis is that all bats with known sequences have aspartate instead of glutamate in a specific locus in IIS3-S4 (Fig 2D). The functional implications of this substitution are presently unclear. At least one other bat species, Hemprich’s Long-eared bat (*Otonycteris hemprichii)*, is resistant to scorpion venom [5]. This species is also a gleaning bat found in the Negev desert, where it is observed to hunt the highly venomous Deathstalker scorpion (*Leirus quinquestriatus)* [5]. A few studies have documented scorpion parts in the diet of other bats [46–49], but identities of these scorpions are not known. Future comparative analyses of sodium ion channel sequences from bats that hunt scorpions *versus* aerial hawking bats will inform studies of evolution of venom resistance and gleaning behavior in bats.

While IVS5-S6 is not a known venom toxin-binding region, altered amino acid side chain charge highlighted in Fig 2E could alter toxin binding allosterically. For example, in venom susceptible animals, two consecutive lysine residues (K1705 and K1706) occur adjacent to valine at V1707. The K1705E and V1707D substitutions in the pallid bat result in a local charge alteration from to +2 in venom susceptible animals to -1. This is the same type of substitution seen in naked mole rats Nav1.7, which reduces nociceptor firing in response to acidic conditions [50]. Taken together, these differences in chemical properties become compelling targets for functional analysis. Although the focus here has been on sodium ion channels, other mechanisms of venom resistance could include neutralization of toxic proteases/phospholipases by inhibitors in pallid bat blood, as has been seen in other species [51]. Future studies will mix bat serum with venom for injection into mice to determine if this mechanism is involved in venom resistance.

### Conclusions

This study presents the first evidence that pallid bats are resistant to Arizona bark scorpion venom at concentrations that causes significant pain and death in mice. Sequencing of the voltage gated sodium ion channels present promising sites to begin investigating precise mechanisms that confer venom resistance. Some of these changes are confined to the pallid bat, while others are observed across the various bat species examined. Future investigations will focus on regional differences in venom tolerance, functional consequences of sodium channel sequence alterations for pain tolerance, and amino acid substitutions seen in bats that may have been subject to positive selection. Together these data indicate that the pallid bat has evolved novel mechanisms of pain modulation involving altered ion channel function.

## Supporting information

S1 Video Bat scorpion encounter. Video showing pallid bat being stung at least twice by an Arizona bark scorpion during a hunt.
(MP4)

S1 Fig Trinity run. Information on Trinity run used to assemble the transcriptome.
(PNG)

S2 Fig Nav1.8 alignments. Alignment of pallid bat Nav1.8 with select species.
(TIF)

S1 Table Assembly statistics. Information on Trinity run used to assemble the transcriptome.
(DOCX)
