# Supplementary figures and images for: Arizona bark scorpion venom resistance in the pallid bat, Antrozous pallidus
Source: PLoS One. 2017 Aug 30;12(8):e0183215. doi: 10.1371/journal.pone.0183215 (PMC5576675; doi:10.1371/journal.pone.0183215)

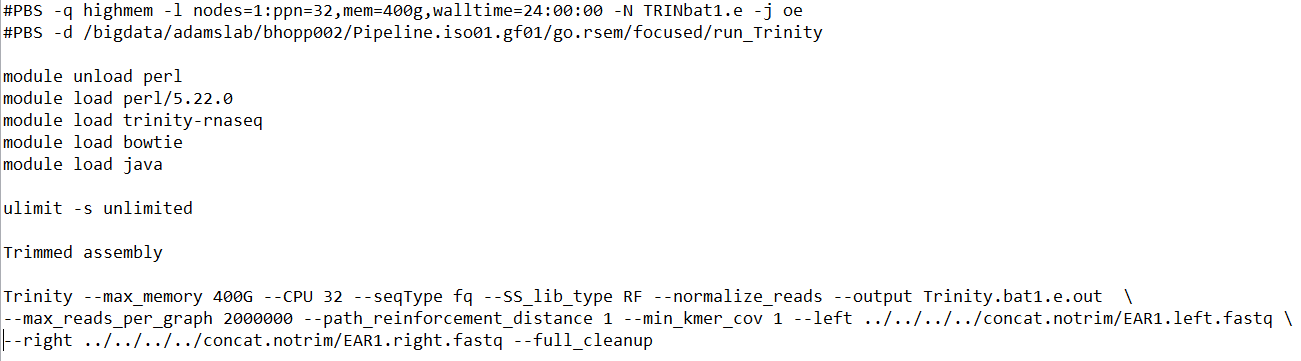

Supplement: S1 Fig — Information on Trinity run used to assemble the transcriptome. (PNG) [file pone.0183215.s002.PNG]

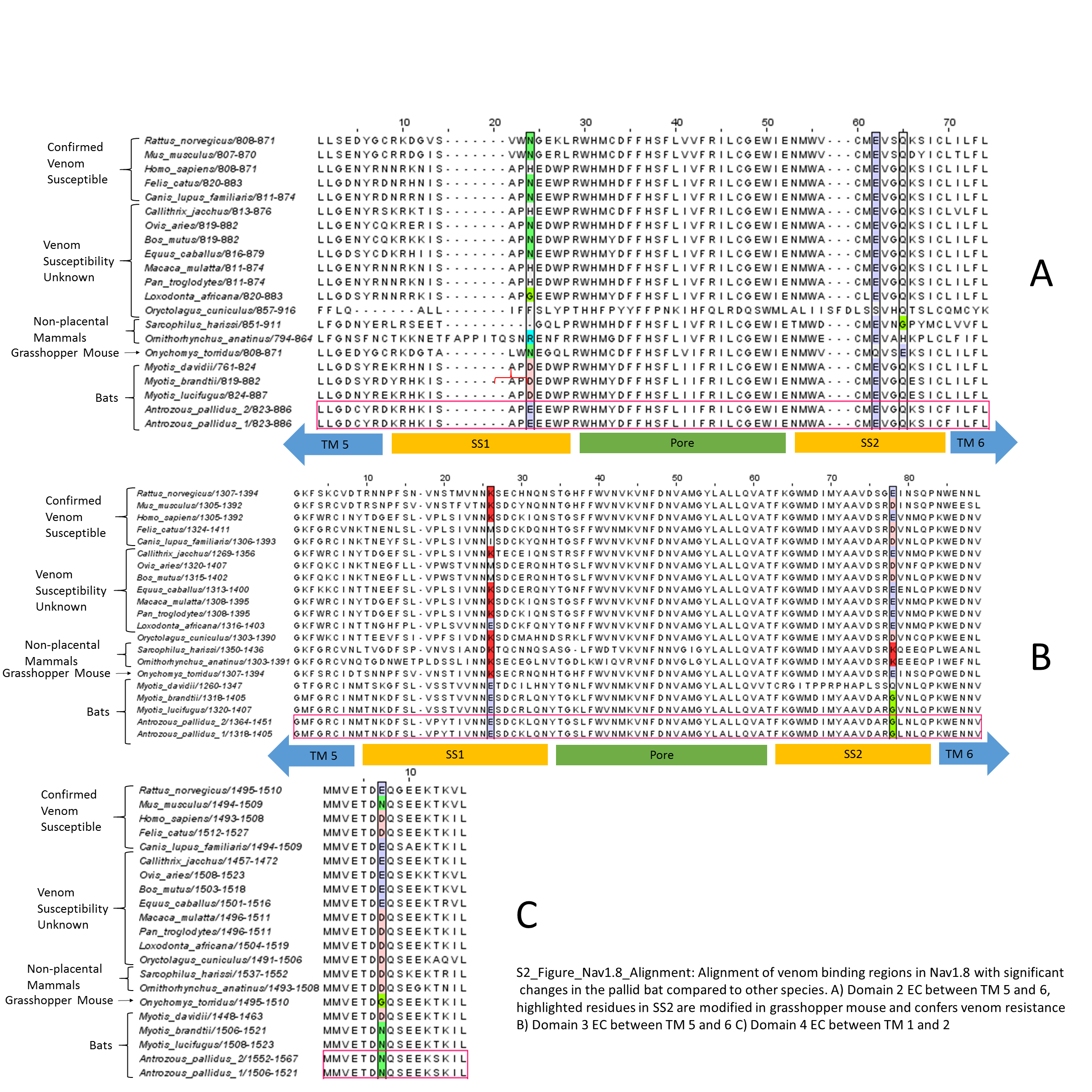

Supplement: S2 Fig — Alignment of pallid bat Nav1.8 with select species. (TIF) [file pone.0183215.s003.tif]
