# Supplementary material for: Arizona bark scorpion venom resistance in the pallid bat, Antrozous pallidus
Source: PLoS One. 2017 Aug 30;12(8):e0183215. doi: 10.1371/journal.pone.0183215 (PMC5576675; doi:10.1371/journal.pone.0183215)
Supplement: S1 Table — Information on Trinity run used to assemble the transcriptome. (DOCX) [file pone.0183215.s004.docx]

| **DRG1 Assembly** | |  | **DRG2 Assembly** | |
| --- | --- | --- | --- | --- |
| ######## |  |  | ######## |  |
|  |  |  |  |  |
| # Transfrags | ######## |  | # Transfrags | ######## |
| Median Transfrag | 342.00 |  | Median Transfrag | 323.00 |
| Average Transfrag Length | 797.53 |  | Average Transfrag Length | 767.62 |
| N50 | 1,695.00 |  | N50 | 1,683.00 |
|  |  |  |  |  |
| **BUSCO** |  |  | **BUSCO** |  |
| Number found | 338.00 |  | Number found | 350.00 |
| Number partial | 77.00 |  | Number partial | 53.00 |
| Number not found | 30.00 |  | Number not found | 24.00 |
|  |  |  |  |  |
|  |  |  |  |  |
| **TransDecoder** | |  | **TransDecoder** | |
| Number ORFS | 94,522.00 |  | Number ORFS | ######## |
|  |  |  |  |  |
| DRG1 |  |  | DRG2 |  |
| **Myotis Lucifugus Run** | | | **Myotis Lucifugus Run** | |
| Number Hits | 64,028 |  | Number Hits | 70,783 |
| Number Unique Hits | 13,255 |  | Number Unique Hits | 13,667 |
|  |  |  |  |  |
| **Swissprot Run** | |  | **Swissprot Run** | |
| Number Total Hits | 66,401 |  | Number Total Hits | 73,190 |
| Number Unique Gene Hits | 13,807 |  | Number Unique Gene Hits | 14,346 |
